# Promoter polymorphism -119C/G in *MYG1 *(C12orf10) gene is related to vitiligo susceptibility and Arg4Gln affects mitochondrial entrance of Myg1

**DOI:** 10.1186/1471-2350-11-56

**Published:** 2010-04-08

**Authors:** Mari-Anne Philips, Külli Kingo, Maire Karelson, Ranno Rätsep, Eerik Aunin, Ene Reimann, Paula Reemann, Orm Porosaar, Jonas Vikeså, Finn C Nielsen, Eero Vasar, Helgi Silm, Sulev Kõks

**Affiliations:** 1 Department of Physiology, University of Tartu, 19 Ravila Street, Tartu 50411, Estonia; 2 Department of Dermatology and Venerology, University of Tartu, 31 Raja Street, 50417 Tartu, Estonia; 3 Department of Paediatric Surgery, Tallinn Children's Hospital, 28 Tervise Street, 13419 Tallinn, Estonia; 4 Department of Clinical Biochemistry, Rigshospitalet, University of Copenhagen, DK-2100 Copenhagen, Denmark

## Abstract

**Background:**

*MYG1 *(*Melanocyte proliferating gene 1*, also C12orf10 in human) is a ubiquitous nucleo-mitochondrial protein, involved in early developmental processes and in adult stress/illness conditions. We recently showed that *MYG1 *mRNA expression is elevated in the skin of vitiligo patients. Our aim was to examine nine known polymorphisms in the *MYG1 *gene, to investigate their functionality, and to study their association with vitiligo susceptibility.

**Methods:**

Nine single nucleotide polymorphisms (SNPs) in the *MYG1 *locus were investigated by SNPlex assay and/or sequencing in vitiligo patients (n = 124) and controls (n = 325). *MYG1 *expression in skin biopsies was detected by quantitative-real time PCR (Q-RT-PCR) and polymorphisms were further analysed using luciferase and YFP reporters in the cell culture.

**Results:**

Control subjects with -119G promoter allele (rs1465073) exhibited significantly higher *MYG1 *mRNA levels than controls with -119C allele (*P *= 0.01). Higher activity of -119G promoter was confirmed by luciferase assay. Single marker association analysis showed that the -119G allele was more frequent in vitiligo patients (47.1%) compared to controls (39.3%, *P *< 0.05, OR 1.37, 95%CI 1.02-1.85). Analysis based on the stage of progression of the vitiligo revealed that the increased frequency of -119G allele occurred prevalently in the group of patients with active vitiligo (n = 86) compared to the control group (48.2% *versus *39.3%, *P *< 0.05; OR 1.44, 95%CI 1.02-2.03). Additionally, we showed that glutamine in the fourth position (in Arg4Gln polymorphism) completely eliminated mitochondrial entrance of YFP-tagged Myg1 protein in cell culture. The analysis of available EST, cDNA and genomic DNA sequences revealed that Myg1 4Gln allele is remarkably present in human populations but is never detected in homozygous state according to the HapMap database.

**Conclusions:**

Our study demonstrated that both *MYG1 *promoter polymorphism -119C/G and Arg4Gln polymorphism in the mitochondrial signal of Myg1 have a functional impact on the regulation of the *MYG1 *gene and promoter polymorphism (-119C/G) is related with suspectibility for actively progressing vitiligo.

## Background

Vitiligo is an acquired pigmentary disorder characterized by areas of depigmented skin resulting from loss of epidermal melanocytes [1]. Considered the most common pigmentary disorder, vitiligo occurs with a frequency of 0.1-2.0% in various populations [2]. Strong evidence from twin and family studies indicates the importance of genetic factors in the development of vitiligo [1]. Vitiligo involves complex interaction of environmental and genetic factors that ultimately contribute to melanocyte destruction, resulting in characteristic depigmented lesions [3]. Recently we have proposed a novel gene, *MYG1 *(*Melanocyte proliferating gene 1*) to be involved in vitiligo genetics. We have shown elevated expression of *MYG1 *mRNA in both uninvolved and involved skin in case of vitiligo [4]. Additionally we have shown that Myg1 is ubiquitously expressed with subcellular localization in the mitochondria and nucleus. *MYG1 *has differential pattern and level of expression during embryonic development of mouse [5]; however, *MYG1 *expression in normal adult tissues is stable and seems to be changed mainly as a response to stress/illness conditions [4,6,7]. Recently, *MYG1 *has been found to be consistently up-regulated also in skin biopsies from patients with atopic eczema [8] that is a common inflammatory skin disorder. Thus elevated *MYG1 *mRNA expression has been described in two dermatological diseases.

The link between vitiligo and human 12q13 locus that includes *MYG1 *has been recently reported. *VDR *gene encoding vitamin D receptor in 12q13 locus associates with vitiligo in a small inbred Romanian community [9]. However, the *VDR *and *MYG1 *genes are separated by 5.394 Mb and these two genes are not linked. The precise function of Myg1 in the development of vitiligo is not clear. However, up-regulation of several immune response-related genes after siRNA-mediated knockdown of *MYG1 *mRNA suggests that Myg1 may participate in pathways proposed by autoimmune theory of vitiligo pathogenesis [3]. Mitochondrial localization of Myg1 fits also with alternative theory of altered mitochondrial functionality of vitiligo patients [10,11].

In human, the *MYG1 *gene (also known as C12orf10) is composed of seven exons that span 7.5 kb of genomic DNA in chromosomal region 12q13. According to NCBI's dbSNP database http://www.ncbi.nlm.nih.gov/SNP/ the *MYG1 *gene contains 10 polymorphisms that are defined as single nucleotide polymorphisms (SNPs). None of the *MYG1 *polymorphisms have been previously studied in association analysis, but two polymorphisms are potentially functional. SNP rs1465073 is located 119 bp upstream of *MYG1 *translation start site (ATG) and we hereafter designate this SNP as *MYG1 *promoter polymorphism (-119C/G). Another polymorphism involves nucleotides 11-12 (rs1534284-rs1534283) downstream from translation start ATG. As it becomes evident from EST and genomic DNA sequence analysis, these polymorphisms are not real SNPs because they always appear as couple AA or GC (Table 1) in human populations. These nucleotides are coding second and third position of amino acid four in the N-terminus of Myg1 protein (CAA and CGC, respectively). According to our previous study, amino acid four is part of a mitochondrial targeting signal (MTS) of Myg1 protein and the polymorphism is potentially functional, since it changes basic amino acid into polar and uncharged. CGC that is common in Caucasians codes for basic amino acid arginine (Arg); CAA that according to the HapMap database http://www.hapmap.org[12] is highly prevalent in the Nigerian population is coding for polar and uncharged amino acid glutamine (Gln). We hereafter refer to rs1534283-rs1534284 polymorphism as Myg1 Arg4Gln.

**Table 1:** Analysis of rs1534283 and rs1534284 variants (Myg1 Arg4Gln) in human EST (mRNA) and genomic DNA sequences from NCBI and UCSC databases.

	Fourth amino acid	Database/Accession	Sequence coding eight first amino acids from *MYG1 *initial ATG	Source of mRNA
**mRNA sequences**	Gln	[GenBank:NM_021640]	atgggacaccaattcctgcgcggc	
	Gln	[GenBank:CR626228.1]	atgggacaccaattcctgcgcggc	fetal brain
	Gln	[GenBank:CR614390.1]	atgggacaccaattcctgcgcggc	neuroblastoma
	Arg	[GenBank:BC051871]	atgggacaccgcttcctgcgcggc	
	Arg	[GenBank:BC013956]	ccgcttcctgcgcggc	
	Arg	[GenBank:AF289485]	atgggacaccgcttcctgcgcggc	melanocytes
				
**Genomic DNA**	Gln	[GenBank:NT_029419]	ATGGGACACCAATTCCTGCGCGGC	
	Gln	[GenBank:NC_000012]	ATGGGACACCAATTCCTGCGCGGC	
	Gln	UCSC database	ATGGGACACCAATTCCTGCGCGGC	
	Arg	[GenBank:NW_925395.1]	ATGGGACACCGCTTCCTGCGCGGC	

First (ATG) and fourth (CAA or CGC) amino acids are underlined.

The purpose of the present study was to examine nine known SNPs in the *MYG1 *gene selected from the HapMap database and to study their associations with vitiligo susceptibility. Moreover, we studied a possible relationship between *MYG1 *promoter SNP (-119C/G) and the amount of *MYG1 *mRNA being transcribed. Finally our purpose was to characterize potentially functional *MYG1 *polymorphisms further using cell culture experiments.

## Methods

### Characteristics of study participants

Unrelated Caucasian patients living in Estonia with a clear clinical diagnosis of vitiligo (n = 124, 88 female, 36 male, age range 18-82 years, mean age of onset of vitiligo 27.8 years) were enrolled at the Department of Dermatology, University of Tartu, Estonia. The diagnosis of vitiligo was based on such clinical signs as characteristic skin depigmentation with typical localization and white colour on the skin lesions under Woods lamp. The clinical types of vitiligo were classified as focal (one or few macules in a nondermatomal distribution; n = 5), segmental (unilateral segmental distribution; n = 4), acrofacial (distal extremities and face; n = 11), vulgaris (scattered over the body; n = 101) or universal (over 90% depigmentation; n = 3). Based on the stage of progression of the disorder, patients were divided into two subgroups: patients with active vitiligo (in which new lesions appeared and existing lesions increased in size over the past 3 months; n = 86) and patients with stable vitiligo (in which depigmentation did not increase during the last 3 months; n = 38). Patients were also divided into four subgroups based on the extent of the skin lesion: less than 10% (n = 65); 10-50% (n = 37); 51-90% (n = 15) and more than 90% (n = 3). 46 patients (37.1%) had one or more disease of autoimmune origin including autoimmune thyreoiditis, rheumatoid arthritis and diabetes, psoriasis, alopecia areata and pernicious anemia. The control group consisted of 325 healthy unrelated Caucasians (180 female, 145 male, age range 18-71 years) without a personal or family history of vitiligo as described earlier [4]. The Ethics Review Committee on Human Research of the University of Tartu approved the study and written informed consent was obtained from all participants.

### Detection and statistical analysis of the human *MYG1 *gene polymorphisms

**Figure 1:**
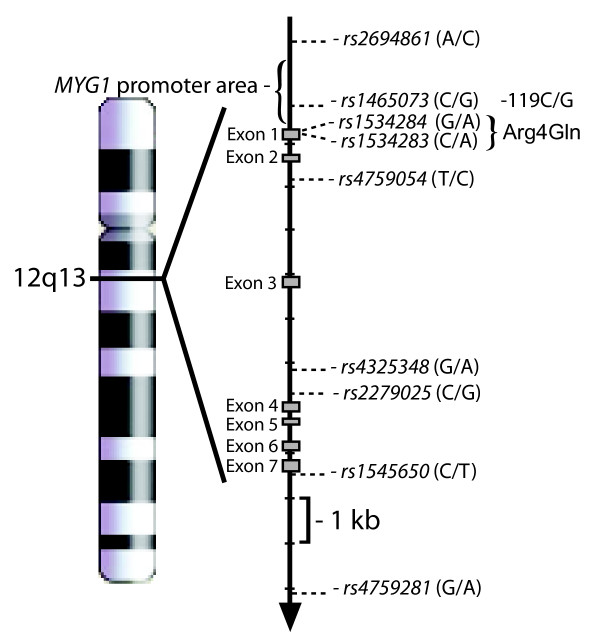
**Genomic localization of single nucleotide polymorphisms (SNPs) in the *MYG1 *gene that were examined in this study**. Relative positions of selected SNPs are represented by their ID number from NCBI's dbSNP database. Grey boxes are representing exons and arrow indicates the direction of transcription of *MYG1 *gene.

DNA was extracted from blood samples by standard salting-out method. A panel of nine SNPs located inside and also up- and downstream of the *MYG1 *gene was investigated. The ID numbers from NCBI's dbSNP database of *MYG1 *SNPs that were examined in this study are listed on Figure 1. For the SNPs rs2694861, rs1465073, rs1534284, rs4759054, rs4325348, rs2279025, rs1545650, and 4759281 the Applied Biosystems (Foster City, California) SNPlex assay pool was used. The ZipCode probes were detected with an Applied Biosystems 3730 DNA Analyzer, and data interpretation was performed with the Applied Biosystems Genemapper v4.0 software [13]. SNPbrowser version 3.5 was used for SNP selection and SNPlex assay pool design. Single marker association analysis was performed using the Haploview program [14]. Allele frequencies were investigated using the chi-square test. To evaluate deviation from the Hardy-Weinberg equilibrium, observed and expected genotype frequencies were compared by Fisher's exact test in the examined groups (cases and controls). Pairwise linkage disequilibrium (LD) between markers was estimated by a log-linear model and standardized D' characteristics were used to demonstrate the extent of disequilibrium. Since SNPlex assay was not functional for detecting rs1534284, we used direct sequencing for rs153484 and neighbour SNP rs1534283 in 54 subjects (24 vitiligo patients and 30 healthy controls) using ABI Genetic Analyzer 310 (Applied Biosystems, Foster City, CA, USA). The direct sequencing of incidental DNA samples was also performed for rs1465073 for verification of the SNPlex results.

### Detection of *MYG1 *mRNA levels by real-time PCR

Expression of *MYG1 *mRNA in the skin biopsies of human subjects was measured as described previously and partially samples from our previous study were used [4].

Gene expression level of *MYG1 *was detected applying TaqMan Assay-On-Demand (Hs000222208_m1, FAM-labelled MGB-probe) gene expression assay mix (20×, Applied Biosystems, Foster City, CA, USA) and TaqMan^® ^Universal PCR Master Mix (Applied Biosystems, Foster City, CA, USA) in the ABI Prism 7900 HT Sequence Detection System (Applied Biosystems, Foster City, CA, USA). Reactions were carried out in 10 μl reaction volumes in four replicates. Data is presented as 2^-ΔCT ^calculated in relation to the *HPRT-1 *[15] and statistical analysis was performed by using t-test. For analysis of functional significance of promoter genotype in the regulation of gene expression, we used subjects (n = 52) from whom both genomic DNA and skin biopsy was available. *MYG1 *expression was measured from skin biopsy of 24 normal control subjects (15 female, 9 male) and 28 vitiligo patients (20 female, 8 male).

### *MYG1 *cDNA constructs

Full-length human *MYG1 *cDNA with both variants for Myg1 Arg4Gln polymorphism were inserted into pEYFP-N1 vector (Clontech, Palo Alto, USA) by using NheI and SacII restriction sites. Myg1 4Arg cDNA was cloned by using primers 1 and 3 (see Table 2 for primer sequences) and Myg1 4Gln cDNA with primers 2 and 3. For *MYG1 *promoter analysis, fragments from *MYG1 *promoter area were cloned into pGL3-Basic Vector (Promega) by using KpnI and HindIII restriction sites. Genomic DNA from two control subjects was used to make reporter gene constructs and all fragments were sequenced to verify genotype and lack of additional mutations in the constructs. Short promoter fragment that includes 291 bp genomic sequence before *MYG1 *coding ATG was amplified by using primers 4 and 6; long, 1 kb promoter fragment was amplified with primers 5 and 6. Both long and short promoter fragments were created in two variants with respect of *MYG1 *promoter polymorphism -119C/G. Two different lengths of promoter fragments were used because of lack of information how long promoter area is needed to trigger maximum promoter activity for the *MYG1 *gene. 291 bp fragment was used, because it is the maximum *MYG1 *unique promoter area that is not overlapped with 3'-UTR of *PFDN5*.

**Table 2:** Primer sequences used for cloning DNA constructs.

No	Name	Sequence
1.	Human MYG1 4Arg ATG Fw	5'-ATATgctagcCATGGGACACCGCTTCCTGCGCG-3'
2.	Human MYG1 4Gln ATG Fw	5'-ATATgctagcCATGGGACACCAATTCCTGCGCG-3'
3.	Human MYG1 noSTOP Rev	5'-ATATccgcggAGATTTGTGGGAGGTATGAG-3'
4.	MYG1-300KpnIF	5'-TATAggtaccAGAATGTTGGTCTTTTCTTGGATTAAGC-3'
5.	MYG1-1kbKpnIF	5'-TATAggtaccGAGAAGAGTCTCATTCTCACC-3'
6.	MYG15utrHindIIIR	5'-ATATaagcttAAGCAGCTCCCTGCAGGGAG-3'

First (ATG) and fourth (CAA or CGC) amino acids in primers 1 and 2 are underlined; restriction sites are given in lowercase letters.

### Cell culture experiments and luciferase assay

All cell culture experiments were performed with HeLa cells. *MYG1 *cDNA and promoter DNA constructs were transfected into cells by FuGENE 6 Transfection Reagent (Roche, USA). Mitochondria were visualized by using MitoTracker Red CMXRos (Molecular Probes, USA). Images were acquired with a Zeiss LSM 510 confocal laser-scanning microscope. For luciferase assay 200,000 cells per well were plated into six-well cell-culture dishes 24 h prior transfections and five replicas were created for each plasmid, with two different DNA preparations of the same clone. For each well 1 μg of the construct was transfected along with 1 μg of pRLO renilla luciferase reporter vector as the control for transfection efficiency. Luciferase gene with CMV promoter was used as positive control and empty pGL3-Basic vector as negative control. The cells were harvested 24 h after transfection, and the activity of firefly and renilla luciferase was measured with GloMax 96 Luminometer (Promega). The normalized luciferase data (renilla/firefly) were used to perform statistics (t-test) and are expressed relative to empty pGL3-Basic vector.

## Results

### *MYG1 *gene polymorphisms associated with vitiligo susceptibility

From the initial nine SNPs, we failed to genotype rs1534284 and rs4759054 with SNPlex platform. Moreover, four SNPs (rs2694861, rs4325348, rs2279025, and 4759281) were monogenic in Estonian population with only major alleles being present. Sequencing genomic DNA of 54 subjects (30 controls and 24 vitiligo patients) revealed that rs1534284/rs1534283 double-polymorphism is likewise prevalently monogenic in Estonian population with only Myg1 4Arg allele being present. The polymorphic SNPs in our study were rs1465073 in *MYG1 *promoter (-119C/G) and rs1545650 (C/T) located in short 281 bp intergenic area between *MYG1 *and *AAAS *[GenBank:NM_015665] gene. Direct sequencing of rs1465073 polymorphism in 40 incidental subjects completely overlapped with SNPlex results. The minor allele (G) of the rs1465073 was more frequent in the vitiligo group compared to the control group (47.1% *versus *39.3%, *P *= 0.0385; OR 1.37, 95%CI 1.02-1.85) consistent with a susceptibility effect. Analysis based on the stage of progression of the vitiligo revealed that the increased frequency of the minor allele (G) of rs1465073 occurred prevalently in the group of patients with active vitiligo (n = 86) compared to the control group (48.2% *versus *39.3%, *P *= 0.0398; OR 1.44, 95%CI 1.02-2.03). There was no statistically significant effect in the distribution of the minor allele of rs1465073 between patients with stable vitiligo (n = 38) and control group (44.6% *versus *39.3%, *P *= 0.378; OR 1.24, 95%CI 0.76-2.02). The average age, mean onset and duration of vitiligo did not differ between patients with active *versus *stable vitiligo. Although minor allele (T) of rs1545650 was more prevalent in patients than in control individuals (3.3% *versus *1.6%, respectively), the difference remained not significant (*P *= 0.172; OR 2.08; 95% CI 0.71-6.07). LD analysis (solid spine algorithm) indicated that the rs1465073 polymorphism, which is located in the promoter of the *MYG1 *gene, was not in strong LD with the *MYG1 *rs1545650 polymorphism located 7.7 kb downstream (|D'| 0.23). No significant effects in the distribution of the *MYG1 *gene allele frequencies were found when patients with different clinical subtypes of vitiligo were analysed separately or when analysis was performed regarding the extent of the skin lesion. Also, polymorphisms in the *MYG1 *gene were not related with concurrent autoimmune disease in vitiligo patients.

### *In vivo *and *in vitro *studies of *MYG1 *promoter activity

**Figure 2:**
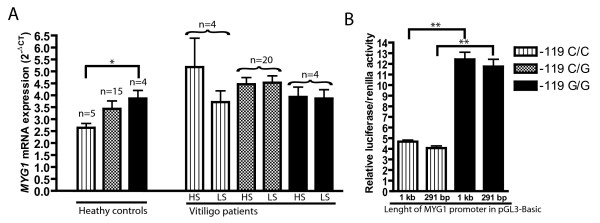
**Impact of *MYG1 *promoter SNP (-119C/G) on *MYG1 *mRNA expression**. (A) *MYG1 *mRNA expression levels in the skin biopsies of healthy subjects and vitiligo patients; n indicates number of subjects in each group; the error bars present standard deviation of the mean. Abbreviations: HS - healthy skin; LS - lesioned skin. (B) *MYG1 *promoter activity in luciferase assay. Data is shown relative to the activity of empty pGL3-Basic. Pattern codes corresponding to three genotypes for both (A) and (B) are shown on the right. * P < 0.05; ** P < 0.01

*MYG1 *mRNA levels in the skin of healthy controls correlated with -119C/G polymorphism (Figure 2A, healthy controls). *MYG1 *mRNA expression of G homozygous subjects (2^-ΔCT ^= 3.87 ± 0.67) was significantly (*P *= 0.0107) higher than in subjects who had homozygous C in the same position (2^-ΔCT ^= 2.64 ± 0.39). The expression value of heterozygous subjects remained in between (2^-ΔCT ^= 3.43 ± 1.29), but due to relatively high deviation, it was not statistically different from either homozygous groups. In vitiligo patients we could not detect a similar difference and there were no statistically significant differences in *MYG1 *mRNA levels between the groups divided according to -119C/G genotypes (Figure 2A, vitiligo patients). Additionally we performed luciferase reporter assay in the cell culture to measure the influence of -119C/G polymorphism in the *in vitro *system with minimum interacting factors. In luciferase assay both short and long promoter fragment with -119G allele had more than 2.5 fold higher activity than -119C allele (Figure 2B). Comparison of 291 bp promoter fragments showed that -119G allele had 2.66 times higher activity (*P *< 0.01) and comparison of 1 kb promoter fragments revealed that -119G allele had 2.89 times higher activity (*P *< 0.01). Additionally our results demonstrate that 291 bp *MYG1 *promoter fragment is sufficient to trigger maximum activity of the *MYG1 *gene. The CMV promoter that we used as a positive control induces high-level constitutive expression in a variety of mammalian cell lines [16]. In general *MYG1 *promoter was 4-8 times less active than CMV promoter depending on the promoter allele (data not shown).

### Arg4Gln polymorphism affects subcellular localization of Myg1 in cell culture

**Figure 3:**
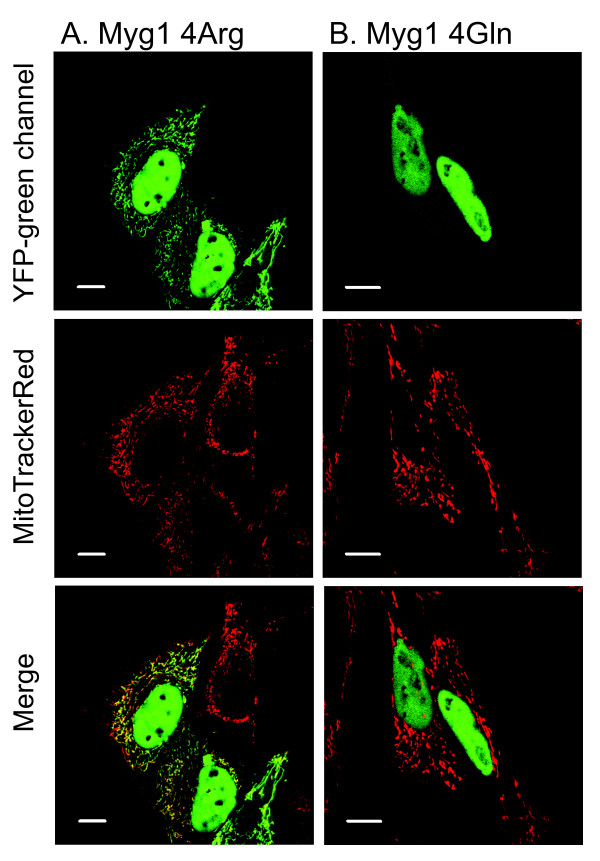
**Subcellular localization of full-length human *MYG1 *cDNA with both variants for Myg1 Arg4Gln polymorphism**. Mitochondrial localization YFP tagged *MYG1 *cDNA with arginine in the position amino acid four (A) is completely eliminated if arginine is replaced with glutamine (B). Scale bars represent 10 μm.

To study if Myg1 Arg4Gln polymorphism has an influence on subcellular localization of Myg1, we expressed both YFP-tagged cDNA variants for Myg1 Arg4Gln polymorphism in HeLa cells. We have previously shown that full-length Myg1 4Arg cDNA that is common among Caucasians localizes into the nucleus and mitochondria [5]. The same localization was confirmed in the current study (Figure 3A). Myg1 4Gln variant was completely unable to enter mitochondria. We could detect YFP signal in the nucleus and slight homogenous signal in the cytoplasm, but there were no cells where Myg1 4Gln-YFP signal would be overlapped with MitoTracker (Figure 3B).

## Discussion

*MYG1 *mRNA expression is elevated in the skin of vitiligo patients [4]. In the present study we showed that *MYG1 *mRNA levels in skin samples of healthy controls correlate with *MYG1 *promoter polymorphism -119C/G and subjects with homozygous -119G allele have significantly higher *MYG1 *mRNA levels than subjects with homozygous -119C allele. Higher activity of -119G promoter was confirmed by using *in vitro *luciferase reporter assay. -119G promoter was on average more than 2.5 fold more active regardless of the length of the promoter fragment. Single marker association analysis showed that the active -119G allele was more frequent in vitiligo patients compared to controls. Further analysis based on the stage of progression of the vitiligo revealed that the increased frequency of more active -119G allele occurred prevalently in the group of patients with active vitiligo compared to the control group. This finding is in line with our previous study [4] where no difference in *MYG1 *expression between nonlesional skin of stable subtype of vitiligo and healthy control subjects was detected. At the same time a statistically significant increase in *MYG1 *expression in both lesional and nonlesional skin of patients with active vitiligo and in lesional skin of patients with stable vitiligo was found, raising the possibility that *MYG1 *is least reactive in the stable stage unaffected vitiligo skin [4]. According to growing evidence, the *MYG1 *gene is predominantly implicated in actively progressing vitiligo.

**Figure 4:**
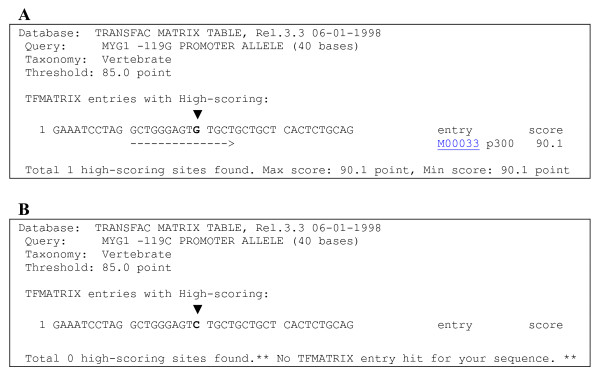
**Analysis of binding sites in the -119 promoter region using TRANSFAC software**. (A) Sequence of -119G promoter allele. (B) Sequence of -119G promoter allele. Arrowhead points to a single nucleotide polymorphism, G in (A) and C in (B).

Minor allele -119G was related to higher *MYG1 *mRNA levels only in control group, but not in vitiligo group. In general, *MYG1 *mRNA is elevated in both involved and uninvolved skin of vitiligo patients despite active or less active promoter genotype. This finding suggests that increased *MYG1 *mRNA level in the skin of vitiligo patients is only partially dependent on endogenous promoter activity and there are other factors besides -119C/G polymorphism that mediate *MYG1 *expression levels in the skin of vitiligo patients. It is likely that part of the effect of increased *MYG1 *expression in vitiligo comes from higher prevalence of naturally more active -119G promoter carriers in vitiligo group. We propose that the elevation of *MYG1 *expression in vitiligo can be both cause and effect depending on the case. Vitiligo patients with -119G promoter have genetic inclination for higher expression and patients with less active promoter genotype harbour other genetic susceptibility loci, but *MYG1 *expression in their skin is still increased as a consequence of cellular changes that characterize vitiligo skin. Alteration of p300 (E1A-associated 300 kDa protein) binding site is a potential reason why *MYG1 *-119G promoter allele is more active in normal control subjects and *in vitro *luciferase assay. The analysis of binding sites in the -119 promoter region was performed by using TRANSFAC software (Figure 4A and 4B). Consensus DNA binding sequence for p300 is 5'-GGGAGTG-3' [17] that corresponds 100% to the bases from -125 to -119 of *MYG1*-119G allele. *MYG1*-119C alters the last position of the binding site for p300 (5'-GGGAGTC-3'). p300 that is mostly known as histone deacetylase can also act as a bridge or scaffold between transcription factors and the basal transcription machinery to enhance the transcription activation [18]. However, besides p300 there can be other transcriptional or epigenetic factors that are sensitive to *MYG1*-119C/G substitution.

Expression of YFP-tagged Myg1 fusion proteins in HeLa cells revealed that glutamine in fourth position (Myg1 4Gln allele) completely eliminated mitochondrial entrance of YFP-tagged Myg1 protein. Our analysis of human ESTs and genomic sequences in NCBI and UCSC http://genome.ucsc.edu/ databases confirm that Myg1 Arg4Gln polymorphism is present in human populations (Table 1). According to currently available data, there can be either arginine or glutamine in the fourth position of Myg1 in humans and here we showed that only positively charged arginine in the fourth position enables mitochondrial entrance. It is likely that acidic glutamine disturbs a common property of mitochondrial targeting sequence to form an amphiphilic helical structure that is essential for the effective transport of a mitochondrial protein [19]. It has been described earlier that single amino acid substitution can completely eliminate the functionality of MTS: single substitution glycine 12 to glutamic acid in a mitochondrial targeting sequence disturbs mitochondrial localization of the human wild-type 8-oxoguanine DNA glycosylase (hOGG1) [20]. Our results provide another example of a single amino acid substitution in mitochondrial signal that eliminates mitochondrial entrance of a protein.

Finally, we sequenced 54 Caucasian subjects for Myg1 Arg4Gln and confirmed that they were all homozygous for Myg1 4Arg allele that corresponds to previous HapMap data for 60 Caucasian subjects (Utah residents with ancestry from Northern and Western Europe). According to HapMap database heterozygosity for Myg1 Arg4Gln polymorphism (rs1534284) has been currently detected only in YRI (Yoruba in Ibadan) population from Nigeria. Among 57 subjects from YRI population who were genotyped for HapMap project 20 subjects (35.1%) were heterozygous for Myg1 Arg4Gln polymorphism but there are no subjects who are homozygous for Myg1 4Gln allele. According to currently available data, Myg1 4Gln allele that disturbs mitochondrial entrance of Myg1 has never been detected in the homozygous state. We propose that persons with Myg1 4Gln variant do not survive or have a health condition that keeps them out from the study groups. High frequency of general heterozygosity (0.175) of Myg1 Arg4Gln can also be a target of natural selection, similarly with 9-amino acid deletion in SLC4A1 gene that is completely lethal in the homozygous state [21], but heterozygosity persists with a maximum frequency of 0.175 due to a protective effect with respect to cerebral malaria in Southeast Asia. The frequency of heterozygosity (Myg1 Arg4Gln) in Nigeria is similar to 9-amino acid deletion in the SLC4A1 gene in Asia (0.175). Therefore it is likely that Myg1 Arg4Gln is a target of similar selection. Considering our present subjects, we cannot explain the relevance of our finding in vitiligo, but we propose that Myg1 has crucial functions inside mitochondria that may be implicated in vitiligo.

The expression pattern of Myg1 is ubiquitous, but there is evidence that Myg1 function in the skin can be differential, for example, during embryonic development, *Myg1 *is predominantly expressed in the ectoderm derived tissues, including epidermis [5], indicating that Myg1 is involved in the development of skin, therefore Myg1 function in the skin can be specific at least in some phases of development. Our association analysis and expression studies suggest that *MYG1 *is one of the genes that in interaction with other genetic and environmental factors is responsible for the development of vitiligo. However, *MYG1 *expression in the skin seems to be not specific to melanocytes: according to our preliminary results, *MYG1 *expression in cultured melanocytes is lower than expression in full skin biopsy and comparable or equal with *MYG1 *expression in cultured fibroblasts derived from the same skin (data not shown). Therefore the precise function of *MYG1 *in the development of vitiligo is still unclear but we have two major hypotheses: up-regulation of several immune system-related genes after *MYG1 *siRNA knockdown in cell culture [5] suggests that Myg1 can act as a mediator in the immune processes that are disturbed in vitiligo patients. Several theories have been proposed about the mechanism of vitiligo pathogenesis but autoimmune hypothesis is the most popular at present [22] and a recent comprehensive review about vitiligo genetics [3] emphasizes that genetic factors underlying autoimmune diseases and vitiligo are often overlapping. Besides immunological approach, the study of the metabolic deregulations leading to toxic damage of the melanocytes appears to be more and more relevant [23]. Therefore, alternatively: mitochondrially localized Myg1 can be involved in the regulation of altered metabolism and imbalance of antioxidants in vitiligo. Growing amount of data provides further evidence for an altered mitochondrial functionality of vitiligo patients [10,11]. Different authors suggest that oxidative stress plays a central role in the process of melanocyte degradation [10,23-25]. The fact that *MYG1 *expression is elevated in both uninvolved and involved skin in case of vitiligo is in line with findings that melanocytes from normally pigmented skin of vitiligo patients also exhibit high susceptibility to chemical and physical oxidative stress [23]. Further studies will be needed to explain the mechanisms of vitiligo pathogenesis and to understand the precise function of Myg1 in vitiligo.

## Conclusions

Our *in vivo *and *in vitro *promoter activity analysis together with association analysis confirmes that -119C/G polymorphism influences *MYG1 *mRNA levels. Our results suggest that more active -119G is the risk-allele for the development of vitiligo and more specifically risk-allele for the maintenance of the active progression stage of the disease. We also showed that Myg1 4Gln allele that exists only in heterozygous state in humans disturbs mitochondrial entrance of Myg1. Because our subjects were consistently homozygous for Myg1 4Arg allele, we cannot confirm the relevance of Myg1 Arg4Gln in vitiligo, but our results suggest that Myg1 has indispensable functions in the mitochondria and might be implicated in mitochondrial damage often present in vitiligo patients. Taken together, our study demonstrated that both *MYG1 *promoter polymorphism -119C/G and Arg4Gln polymorphism in the mitochondrial signal of Myg1 have a functional impact on the regulation of the *MYG1 *gene.

## Competing interests

The authors declare that they have no competing interests.

## Authors' contributions

MAP designed and performed cell culture experiments, designed and prepared DNA plasmids, interpreted the results and wrote the draft of the manuscript. KK performed statistical analysis and participated in drafting the manuscript. KK and MK confirmed the diagnosis of vitiligo patients and coordinated the collection of blood and skin samples of the study participants. KK and SK designed SNPlex assay pool. KK, RR and ER prepared DNA samples for genotyping and participated in genotyping studies. EA and PR performed the Q-RT-PCR analysis. PR, OP and JV participated in cell culture experiments and JV made confocal microscopy images. FCN, EV, HS, SK conceived the study, participated in its coordination and draft revision. All authors have read and approved the final manuscript.

## Pre-publication history

The pre-publication history for this paper can be accessed here:

http://www.biomedcentral.com/1471-2350/11/56/prepub
